# Brachial artery blood flow dynamics during sinusoidal leg cycling exercise in humans

**DOI:** 10.14814/phy2.13456

**Published:** 2017-10-09

**Authors:** Yoshiyuki Fukuba, Masako Y Endo, Ayaka Kondo, Yuka Kikugawa, Kohei Miura, Hideaki Kashima, Masaki Fujimoto, Naoyuki Hayashi, Yoshiyuki Fukuoka, Shunsaku Koga

**Affiliations:** ^1^ Department of Exercise Science & Physiology, School of Health Sciences Prefectural University of Hiroshima Hiroshima Japan; ^2^ Graduate School of Decision Science and Technology Tokyo Institute of Technology Tokyo Japan; ^3^ Faculty of Health and Sports Science Doshisha University Kyoto Japan; ^4^ Applied Physiology Laboratory Kobe Design University Kobe Japan

**Keywords:** Blood flow dynamics, brachial artery, forearm skin blood flow, inactive limb, sinusoidal exercise

## Abstract

To explore the control of the peripheral circulation of a nonworking upper limb during leg cycling exercise, blood flow (BF) dynamics in the brachial artery (BA) were determined using a sinusoidal work rate (WR) exercise. Ten healthy subjects performed upright leg cycling exercise at a constant WR for 30 min, followed by 16 min of sinusoidal WR consisting of 4‐min periods of WR fluctuating between a minimum output of 20 W and a maximum output corresponding to ventilatory threshold (VT). Throughout the protocol, pulmonary gas exchange, heart rate (HR), mean arterial blood pressure (MAP), blood velocity (BV), and cross‐sectional area of the BA, forearm skin BF (SBF), and sweating rate (SR) were measured. Each variable was fitted to a sinusoidal model with phase shift (*θ*) and amplitude (A). Nearly all variables closely fit a sinusoidal model. Variables relating to oxygen transport, such as *V*O_2_ and HR, followed the sinusoidal WR pattern with certain delays (*θ*:* V*O_2_; 51.4 ± 4.0°, HR; 41.8 ± 5.4°, mean ± SD). Conversely, BF response in the BA was approximately in antiphase (175.1 ± 28.9°) with a relatively large A, whereas the phase of forearm SBF was dissimilar (65.8 ± 35.9°). Thus, the change of BF through a conduit artery to the nonworking upper limb appears to be the reverse when WR fluctuates during sinusoidal leg exercise, and it appears unlikely that this could be ascribed exclusively to altering the downstream circulation to forearm skin.

## Introduction

During dynamic exercise, blood flow (BF) to the active skeletal muscles and the exercising limb (mainly to the active skeletal musculature, but also to skin and bone) increases in accordance with the oxygen (O_2_) demand and work rate (WR). Active muscles receive the largest portion of the increased blood supply from the systemic circulation. This redistribution of cardiac output also involves a reduction in peripheral circulation to nonexercising tissues, including internal organs (Rowell et al. 1964; Rowell 1986; Calbet et al. 2007; Endo et al. 2008) and inactive musculatures (Blair et al. 1961; Bevegard and Sheperd 1966; Hohimer et al. 1983; Armstrong et al. 1987; Musch et al. 1987; Tanaka et al. 2006), which may also have a role in maintaining mean arterial blood pressure (MAP) in situations of increased vascular conductance (VC), which occur mainly as a result of vasodilation in large active muscles (Ichinose et al. 2008).

The results of some recent human studies, however, have been inconsistent with this concept. Several studies have found an increase, rather than a decrease, in BF to the inactive limb with the WR of exercise of the contralateral limb (in general, BF of brachial artery [BA] in the inactive upper limb during leg exercise) (Green et al. 2002; Tanaka et al. 2006; Padilla et al. 2011a; Simmons et al. 2011; Smith et al. 2014). Recent studies have demonstrated a biphasic response in BF in the BA during prolonged leg cycling exercise at a constant WR (Padilla et al. 2011b; Simmons et al. 2011; Smith et al. 2014). According to one study, forearm BF and its VC decreased transiently during the first few minutes after exercise onset, but subsequently increased beyond the baseline level and the elevated BF in the BA resulted at least partially from thermoregulatory cutaneous vasodilation following prolonged exercise (Simmons et al. 2011). Such BF elevation in the arms during the continuation of dynamic leg exercise may have induced a favorable shear profile to the endothelium adaptation in inactive vasculatures beyond the active limb (e.g., Padilla et al. 2011a; Green et al. 2017). On the other hand, initiation of dynamic leg exercise transiently elicits suppression of forearm BF (Taylor et al. 1992; Padilla et al. 2011b; Smith et al. 2014). Such suppression is mediated by vasoconstriction resulting from increased muscle sympathetic nervous activity (Blair et al. 1961). During the continuation of exercise, the initial vasoconstriction is overcome by the cutaneous vasodilative response as a result of readjustment to thermoregulatory demands, resulting in elevated forearm BF. Therefore, it remains to be elucidated how BF in the BA would respond to altered WR during prolonged exercise lasting long enough to reach the steady state (plateau) of thermoregulatory cutaneous circulation. In other words, it should be clarified whether early suppression of BF immediately after the onset of exercise would still appear during the continuation of exercise.

We have frequently used a sinusoidal WR forcing function rather than step exercise (i.e., constant WR forcing) to determine cardiorespiratory dynamics during exercise (Fukuoka and Ikegami 1990; Haouzi et al. 1993; Fukuoka et al. 1995, 1997). Compared to a constant WR forcing function, the sinusoidal WR forcing function is continuous, but varies smoothly in WR, so that dynamic properties (especially the phase shift) in almost all physiological variables can be clearly estimated, and those interrelationships (i.e., faster/slower among the traceability of targeted variables) can also be easily determined (Wigertz 1970; Swanson 1990; Whipp and Ward 1990). In addition, using a sinusoidal WR forcing function following prolonged constant exercise (set at mid‐WR between low and peak sinusoidal WRs), compared to the step‐change exercise following it, is advantageous. This is because the total work performed during one cyclic sinusoidal exercise remains the same as the previously performed constant exercise, yet the WR fluctuates continuously. Accordingly, we used a sinusoidal WR forcing function to determine the BF response of the BA to altered WR following 30 min of continued constant exercise, which lasted long enough to reach both the newly increased steady state in BF in BA (Simmons et al. 2011; Smith et al. 2014) and a thermoregulatory state (Yamazaki 2002; Yamazaki and Sone 2003). It is hypothesized that BF in the BA is followed sinusoidally by a WR fluctuation and that its phase shift is similar to that of forearm skin BF (SBF) because BF in the BA is at least partially a result of the thermoregulatory cutaneous vasodilation that follows prolonged exercise (Simmons et al. 2011). To address this hypothesis, we evaluated the BF dynamics of the BA and forearm SBF during a sinusoidal WR forcing function followed by prolonged constant WR exercise.

## Materials and Methods

### Subjects

Eleven healthy young male subjects (19–24 years) volunteered for this study. Each subject underwent an initial examination prior to following the main study protocol. During the initial examination, we attempted to measure the subject's blood velocity (BV) by Doppler ultrasonography (detailed procedure described below). However, we were unable to obtain data from one subject owing to the distance and location between the ultrasound window and the vessel. Therefore, 10 subjects finally participated in the main study. All possible risks associated with the participation in the study were explained, and the subjects provided written informed consent. The study was approved by the Ethics Committee of the Prefectural University of Hiroshima and was undertaken in accordance with the Declaration of Helsinki. Subjects had a sedentary lifestyle, performed no regular endurance training, and did not participate in >2 h of aerobic exercise per week. Their mean (standard deviation, SD) height and weight were 168.4 ± 5.3 cm and 56.4 ± 5.0 kg, respectively.

### Experimental protocols

To test their tolerance limit, subjects initially performed an incremental ramp exercise test at a rate of 20 W/min on an electromagnetically braked cycle ergometer (232c‐XL, Combi Corp., Japan) in a partially recumbent position (approximately 10° behind the vertical upright position) at 60 rpm to estimate ventilatory and gas exchange threshold (VT) and peak oxygen uptake (*V*O_2_). VT was estimated using the V‐slope method (Beaver et al. 1986) and gas exchange criteria were used to detect the breakpoints at which there were systematic increases in the ventilatory equivalent for *V*O_2_ (*V*E/*V*O_2_) and end‐tidal partial pressure of oxygen (P_ET_O_2_), with no concomitant increase in the ventilatory equivalent for CO_2_ output (*V*E/*V*CO_2_) or decrease in end‐tidal PCO_2_ (P_ET_CO_2_) (Whipp 1994). Peak *V*O_2_ was determined during last 30 sec of ramp exercise. All exercise tests were performed in an air‐conditioned laboratory (ambient temperature 22–23°C, relative humidity 45–55%) situated at sea level.

For the main sinusoidal WR exercise period, each subject rested on the ergometer saddle against a backrest for approximately 30 min prior to exercise. Initial resting measurements were obtained during a 4‐min period in which the subject rested in the same position as that used for the ramp exercise; leg cycle ergometer exercise then commenced. An electromagnetically braked ergometer (232c‐XL, Combi Corp., Japan) was able to control the WR second by second via a transport cable connected to an external PC software. Both the arms were placed in a relaxed position on side tables that were set approximately at heart level. As a measure of constant WR exercise, subjects exercised for 30 min at the mean WR of the sinusoidally varying exercise. This was followed by 16 min of sinusoidal WR exercise in 4‐min periods (i.e., four repetitions) which was selected as most common frequency in the previous studies using by sinusoidal WR forcing (Casaburi et al. 1977, 1980; Fukuoka and Ikegami 1990; Haouzi et al. 1993; Yamazaki et al. 1994; Fukuoka et al. 1995, 1997; Yamazaki 2002; Yamazaki and Sone 2003), during which WR fluctuated between a minimum of 20 W and a peak corresponding to VT (approximately 50% of the subject's peak *V*O_2_).

#### Measurements

Ventilatory and gas exchange parameters (*V*E, *V*O_2_, *V*CO_2_, P_ET_O_2_, and P_ET_CO_2_) were determined breath by breath using a computerized metabolic measuring system (Aero‐Monitor, Minato Medical Science, Japan). Prior to each exercise test, the flow sensor and gas analyzers were calibrated by introducing a known volume of air at several mean flow rates and gas mixtures of known composition, respectively. The second‐by‐second time course was calculated for each variable by interpolation of the breath‐by‐breath data.

Beat‐to‐beat heart rate (HR) and MAP obtained from the middle finger of the left hand (Finometer PRO; Finapres Medical Systems, the Netherlands). Previous studies have validated this measurement during exercise (Sugawara et al. 2003; Atkinson et al. 2015). SBF was monitored in the center of the right forearm using a Laser Doppler flowmeter (ALF21; Advance Co., Ltd., Japan) as a measure of red blood cell flux. Forearm skin sweating rate (SR) was determined in an enclosed location by capacitance hygrometry, calculated from the relative humidity and temperature (THP‐B3T; Shinei, Japan) of the air flowing out of a 12.56 cm^2^ capsule at the rate of 1.5 L/min (Ikegawa et al. 1985). The circulatory variables described above were converted into digital data using an AD conversion device and software (PowerLab 8/35, ADInstruments, Colorado Springs, CO) at 1 kHz. Then, second‐by‐second time courses were calculated for each variable by interpolating the beat‐by‐beat or average data.

Beat‐by‐beat BV through the right BA to the distal third of the right inactive upper limb as well as vessel diameter were measured using a pulse‐echo Doppler ultrasound (LOGIQ S6; GE Medical Systems, Japan) and a linear 5.0 MHz probe with an insonation angle below 60°. The diameter of each vessel was measured simultaneously with an imaging frequency of 12.0 MHz. The sample volume was positioned in the center of the vessel and adjusted to cover the full diameter of the BA. For every cardiac cycle, the Doppler tracing was analyzed using integral software to obtain the antegrade and retrograde velocities (mean velocity = antegrade velocity − retrograde velocity) in the BA. BF was calculated from the BV and cross‐sectional area of the vessel, as previously described (Endo et al. 2005, 2008; Koga et al. 2005). Briefly, audio‐range signals for the antegrade and retrograde velocities reflected from the moving blood cells as well as the electrocardiogram (ECG) signal were digitally sampled with a 20‐kHz AD conversion (PowerLab 8/30, ADInstruments, Colorado Springs, CO). Audio‐range signal spectra were processed offline by Doppler signal processing software (using a fast Fourier transfer analysis and a 256‐point Hamming window [12.8 msec each]) to yield instantaneous antegrade and retrograde velocities. Velocity signals were recorded at 100 Hz on a computer system, in addition to the ECG, so that beat‐by‐beat data could be analyzed. Finally, second‐by‐second time courses of antegrade, retrograde, and mean net velocities were calculated by interpolation of the beat‐by‐beat data. B‐mode echo images of the right BA were recorded simultaneously using a hard disk drive video recorder, and the diameter of the vessel was measured with on‐screen calipers. Vessel diameters were summarized at rest every 5 min during the first 30 min of constant WR exercise and at 10‐sec intervals during the 16 min of sinusoidal exercise. In this study, the antegrade, retrograde, and mean BFs are referred to as “BF–BA [antegrade]”, “BF–BA [retrograde]”, and “BF–BA [net]”, respectively, although the mean BF (BF–BA [net]) and its VC in the BA (VC–BA = BF–BA [net]/MAP) are the main variables discussed.

#### Model fitting

In order to determine the dynamic characteristics of each variable relative to sinusoidal exercise, the amplitudes of fluctuations and phase shifts were calculated. Second‐by‐second variables were superimposed every three cycles during the second to fourth sinusoidal exercise periods to fit the sinusoidal model, as follows. The first period was excluded because the response during this period theoretically includes a transient nonsinusoidal component. A sinusoidal model was used to broadly describe the response *y(t)*:

(1) y(t)=M+A×sin[(2π/T)×t−θ]

where *t* = time, *T* = period (of sinusoidal WR, i.e., 240 sec), *M* = mean level, *A* = amplitude, and *θ *= phase shift. Curve fitting was performed using a least squares technique (SigmaPlot ver. 12, Systat Software Inc., San Jose, CA). The square of the correlation coefficient (*R* ^2^) was used as a conventional index of goodness of fit. The ratio of A to M (A/M; ×100%) was defined as the relative amplitude of the response. The sinusoidal model fit was considered successful when *R* ^2^ > 0.5.

### Statistical analysis

Values are expressed as mean ± SD. The time course of changes in each variable was assessed using a one‐way repeated measures analysis of variance (ANOVA). Time ranges were divided into two categories for the ANOVA. First, change during the first 30 min of constant exercise with rest was tested. Second, change throughout the sinusoidal exercise, including the values at the end of constant exercise (i.e., at 30 min), was tested separately. When a significant difference was detected, it was further evaluated using Tukey's post hoc test. Thereafter, the difference(s) among the estimated parameters (*θ* or A/M) of the related variables were tested using one‐way ANOVA, coupled with Tukey's post hoc test where appropriate, to identify homogeneous subsets (i.e., to distinguish between subgroups). Statistical significance was accepted when *P *<* *0.05. All statistical procedures were performed using SPSS version 18.0 for Windows (SPSS Inc., Armonk, NY).

## Results

**Figure 1:**
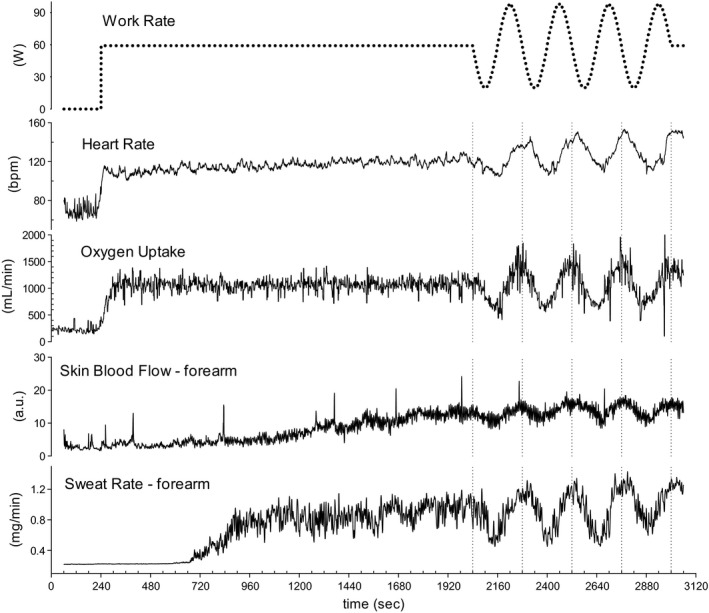
Representative time course of changes in heart rate, O_2_ uptake, skin blood flow, and sweating rate in the forearm during constant work rate exercise (30 min) followed by sinusoidal exercise (16 min; four repetitions each of 4 min) in one subject.

**Figure 2:**
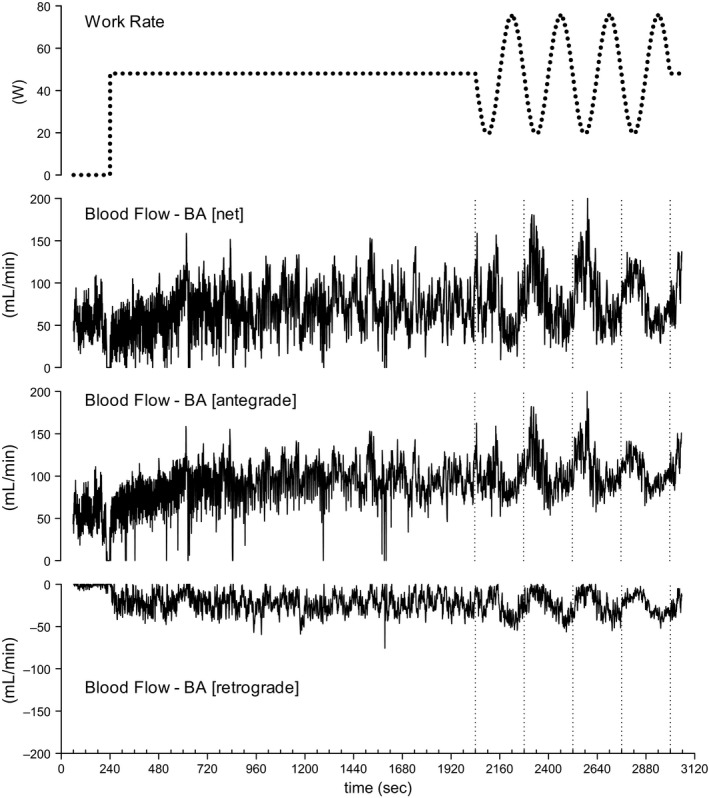
Representative time course of changes in net blood flow in the brachial artery (BA) and its antegrade and retrograde components during constant work rate exercise (30 min) followed by sinusoidal exercise (16 min; four repetitions each of 4 min) in one subject.

The time courses of several principal variables in a representative subject during the study protocol are shown in Figures 1 and 2. During the first 30 min of constant WR exercise, almost all variables showed steady‐state responses, including not only the gas exchange variables, but also SBF and SR during the second half of the constant WR exercise period (at 13–15 min; Table 1). Only HR showed a gradual increase during the 30 min of constant exercise but no further change during the sinusoidal exercise, although mean HR showed a small but significant increase during the fourth wave of the sinusoidal exercise period, compared to 28–30 min of the constant exercise period (Table 1).

**Table 1:** Ventilatory, pulmonary gas exchange, and circulatory measurements at baseline and during constant and sinusoidal work rate exercise periods

		Constant WR exercise		Sinusoidal WR exercise		
	Baseline	13–15 min	28–30 min	2nd	3rd	4th
*V*E, L/min	9.5 ± 1.4	27.1 ± 2.4^a^	27.9 ± 2.3^a^	27.3 ± 2.7	27.3 ± 2.4	27.8 ± 2.5
*V*O_2_, mL/min	211 ± 22	978 ± 70^a^	986 ± 57^a^	950 ± 61	952 ± 58	952 ± 62
*V*CO_2_, mL/min	192 ± 21	912 ± 73^a^	917 ± 69^a^	887 ± 73	882 ± 67	888 ± 69
HR, bpm	71.3 ± 12.3	105.9 ± 13.0^a^	110.7 ± 15.5^a^	111.8 ± 15.3	113.4 ± 15.2	114.1 ± 15.7^c^
MAP, Torr	81.8 ± 6.5	91.4 ± 8.8^a^	93.0 ± 8.6^a^	90.0 ± 10.5	92.6 ± 9.6	94.0 ± 10.9
BF–BA [net], mL/min	77.6 ± 36.0	79.0 ± 28.5	99.8 ± 42.7^a^^,^^b^	97.9 ± 41.9	97.4 ± 29.8	101.1 ± 32.1
BF–BA [antegrade], mL/min	79.9 ± 41.2	100.5 ± 39.1^a^	119.2 ± 57.6^a^	117.2 ± 52.5	115.2 ± 37.6	119.5 ± 40.4
BF–BA [retrograde], mL/min	−2.3 ± 0.6	−22.5 ± 5.9^a^	−19.4 ± 5.2^a^	−19.3 ± 6.3	−17.8 ± 7.5	−18.4 ± 5.9
BA diameter, cm	0.371 ± 0.027	0.359 ± 0.029	0.382 ± 0.032^b^	0.383 ± 0.033	0.380 ± 0.030	0.383 ± 0.028
SBF‐forearm, a.u.	2.7 ± 1.3	10.5 ± 3.5^a^	10.3 ± 4.0^a^	10.9 ± 3.7	11.0 ± 3.6	10.9 ± 3.7
SR‐forearm, mg/min	0.05 ± 0.03	0.23 ± 0.15^a^	0.26 ± 0.19^a^^,^^b^	0.27 ± 0.20	0.27 ± 0.21	0.28 ± 0.21

a: versus baseline, b: versus 13–15 min, c: versus 28–30 min (*P* < 0.05).

Values are mean ± SD (second to fourth in sinusoidal WR exercise calculated by the mean data of one cycle).

BF–BA [net], BF–BA [antegrade], BF–BA [retrograde], and SBF and SR in the forearm reached near steady states within the first 15 min of the 30‐min constant WR exercise period, but BF–BA [net] and SR also showed slight but significant additional increases between 13–15 min and 28–30 min. BA diameter increased significantly from the middle (13–15 min) to the end (28–30 min) of the constant exercise period but did not increase further. None of the upper limb variables showed further changes throughout the sinusoidal exercise period beyond those shown by the end of the constant exercise period (Table 1).

**Figure 3:**
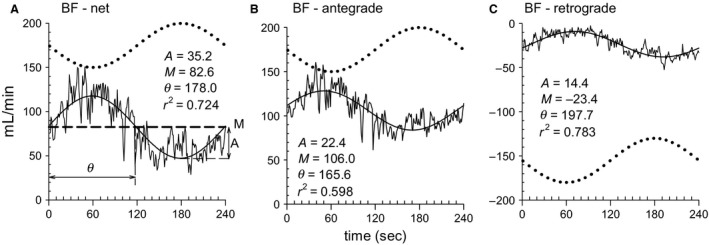
An example of the curve‐fitting analysis. Second‐by‐second responses over the second to fourth sinusoidal exercise cycles were superimposed (net, antegrade, and retrograde BF in the BA) in one subject. The sinusoidal model (solid line) was fitted by the least squares method to estimate the parameters (M, mean; A, amplitude; *θ*, phase lag). *R* ^2^ is a conventional index of goodness of fit. The detailed procedure is described in the Materials and Methods. The dotted line indicates the sinusoidal WR as a reference.

As shown in Figures 1 and 2, the measured variables followed a sinusoidal pattern during the sinusoidal WR exercise period. Therefore, a sinusoidal model with a fixed 4‐min period was fitted to each response. Examples of the model fit for BF in BA are shown in Figure 3. In this instance, Equation (1) was fitted to each net, antegrade, or retrograde component of BV in the BA, using averages of the data by superimposing the second‐by‐second data obtained during the second to fourth sinusoidal exercise periods. The BF [net] and BF [antegrade] responses were nearly sinusoidal (mean *R* ^2^ = 0.640 and 0.701, respectively). The fluctuation in BF [net] appeared like a mirror image response to the sinusoidally varying WR. Some of the subjects showed a sinusoidal response even in BF [retrograde] (Fig. 3C), whereas others demonstrated a poorer fit (mean *R* ^2^ = 0.408). In all likelihood, the latter was mainly a result of zero [retrograde] BF values being recorded during the lower WR phase of one sinusoidal cycle (in 5 of the 10 subjects). BF [retrograde] demonstrated a semicircular profile during the higher WR phase of one sinusoidal cycle in all subjects.

**Figure 4:**
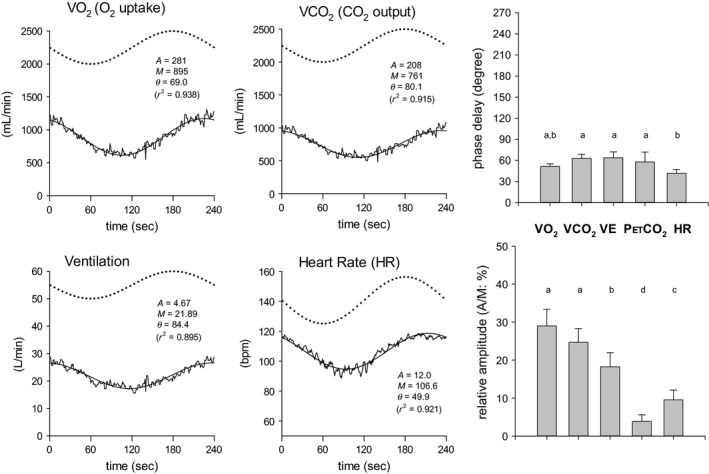
The four panels on the left show examples of superimposed responses (thin lines: *V*O_2_, *V*CO_2_, *V*E, and HR) and fitted sinusoidal models (bold lines). The dotted line indicates the sinusoidal WR as a reference. The panels on the right show the phase delays and relative amplitudes of the principal variables of pulmonary gas exchange and HR. Variables *a*,* b*, and *c* indicate homogeneous data subsets showing no significant differences among them; nonmatching characters indicate *P *<* *0.05 between groups.

**Figure 5:**
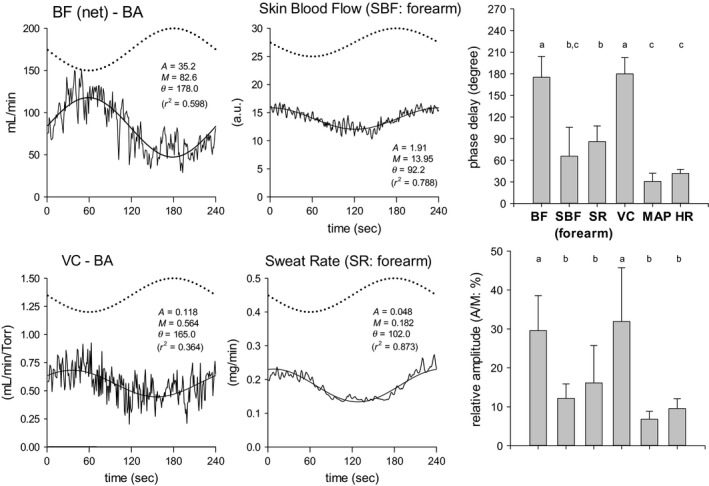
The four panels on the left show examples of superimposed responses (thin lines: BF–BA, VC, and SBF and SR in forearm) and the fitted sinusoidal models (bold line). The dotted line indicates the sinusoidal WR as a reference. The panels on the right show the phase delays and relative amplitudes of the variables of the peripheral upper limb circulation and the systemic circulation. Variables *a*,* b*, and *c* indicate homogeneous data subsets showing no significant differences among them; nonmatching characters indicate *P *<* *0.05 between groups.

The responses in variables describing ventilation, gas exchange, and HR closely followed the sinusoidal WR pattern, accompanied by the expected phase delays (Fig. 4). ANOVA indicated that the phase delay in HR (41.8 ± 5.4°) was significantly less than those of *V*CO_2_ (62.9 ± 5.8°) and *V*E (63.9 ± 8.4°). The phase delay of *V*O_2_ (51.4 ± 4.0°) was between the shorter HR and longer *V*E and *V*CO_2_ responses (Fig. 4). The responses on the other upper limb variables are shown in Figure 5. Both SBF and SR in the forearm also clearly responded in a sinusoidal fashion (mean *R* ^2^ = 0.682 and 0.700, respectively). The VC in BA was quite variable because it was calculated from two physiologically fluctuating parameters but still appeared approximately sinusoidal (mean *R* ^2^ = 0.549).

The phase delays of SBF and SR in the forearm (65.8 ± 35.9° and 85.9 ± 21.7°, respectively) were significantly longer than those of HR and MAP (30.7 ± 11.3°). Furthermore, responses of BF (i.e., BF [net]) and its VC (175.1 ± 28.9°, 179.9 ± 22.6°, respectively) were substantially delayed (Fig. 5). In addition, the relative amplitudes (A/M; %) of BF [net] and VC of BA were ~30% of the mean responses and were significantly higher than those of the other variables (approximately ~7–16%) (Fig. 5). As indicated in Figures 2 and 3, BF to the inactive limb (BF [net]) was in antiphase to the sinusoidal WR pattern but had a relatively larger amplitude.

## Discussion

During sinusoidal WR exercise below the VT (i.e., moderate exercise domain), we observed that: (1) almost all physiological responses followed a sinusoidal pattern; (2) phase shifts in the ventilatory, gas exchange, and systemic circulatory responses were ~30–60°; (3) phase shifts in the SBF and SR in an inactive forearm showed similar but slightly slower responses (~65–85°); and (4) phase shifts in the BF response in the BA and its VC showed antiphasic sinusoidal patterns (~180° phase shift) with a relatively large amplitude. These results were inconsistent with the hypothesis that the phase shift of the BF in the BA to sinusoidal WR fluctuation would be similar to that of the forearm SBF.

Evidence is rapidly accumulating that aerobic exercise training using the lower limb, such as cycling and walking, induces a favorable vascular adaptation even in inactive limbs (Kingwell et al. 1997; Green et al. 2008; Padilla et al. 2011a). However, the vascular mechanisms, whereby such nonspecific or systemic effects occur in the inactive upper limb of individuals undergoing leg exercise training, have not been fully elucidated (Padilla et al. 2011a). Acute exercise‐induced vascular adaptation is essential to obtain the maximum benefit from exercise training. At present, the most plausible candidate mechanism is that the oscillation of BF – the profile of BF in an inactive limb during exercise – may have substantial effect(s) through adaptations of the endothelium (Laughlin et al. 2008). Therefore, the present results contribute to the literature by providing insight into the mechanism(s) underlying an acute vascular adaptation by the detailed BF dynamic properties in the inactive upper limb during a single bout of leg exercise.

Sinusoidal WR forcing exercise is not a new approach to the study of the ventilatory, gas exchange, and HR dynamics during exercise in the sub‐VT domain (Casaburi et al. 1977, 1980; Fukuoka and Ikegami 1990; Fukuoka et al. 1995, 1997) or even in the supra‐VT domain (Haouzi et al. 1993). For example, during 4 min of sinusoidal WR fluctuation in the sub‐VT exercise domain, using a protocol similar to that used in the present study, the phase delays in *V*E, *V*O_2_, *V*CO_2_, P_ET_CO_2_, and HR in five healthy subjects were reported to be 56–83°, 42–60°, 53–76°, 39–75°, and 24–66°, respectively (Casaburi et al. 1977). As shown in Figure 4, responses in the present study were consistent with those reported in previous studies (Fukuoka and Ikegami 1990; Fukuoka et al. 1995, 1997), despite the duration of constant WR exercise prior to the sinusoidal exercise in previous studies being very brief (4–6 min) compared with that of the present study (30 min). To date, the dynamic characteristics of BF in the inactive limb during forced sinusoidal WR exercise have never been elucidated. This was, therefore, the first study to explore the dynamic properties of the BF response in the BA during sinusoidal WR leg cycling. We demonstrated an approximately antiphasic response to altered WR that was quite different from those of the other principle variables of O_2_ transport such as gas exchange and systemic circulation, which showed phase shifts of roughly 30° to 80° (Figs. 1, 2, 4, and 5). This is a major novel finding of the present study.

The response of the BF–BA is determined by both MAP and peripheral VC. The antiphasic response of the BF in the BA appeared to be principally peripheral in origin, because the VC of the BA showed a similar antiphase shift and a consistent large amplitude, while those of the MAP were dissimilar, and instead similar to the changes in HR (Fig. 5). This implies that the changes in BF–BA during one cycle of sinusoidally changed WR exercise were mainly associated with concomitant vasodilation/vasoconstriction of the resistance vessels within the forearm and hand. The BA supplies blood to the skin and skeletal musculatures in the forearm and hand. A huge area of skin in the upper limb distal to the elbow (the forearm and dorsal hand) is nonglabrous where the thermoregulatory function is substantially exerted, and we, therefore, measured the forearm SBF continuously during the study. The phase shift of the forearm SBF was approximately 65°, which was not compatible with that of BF–BA (Fig. 5). To avoid contamination of the developed nonsteady‐state thermoregulatory response during the continuation of exercise, the protocol in the present study was designed to incorporate the preceding 30‐min period of constant exercise at an intermediate WR of the subsequent sinusoidal WR fluctuation before commencing the sinusoidal WR forcing function. As a result, the forearm SBF and SR reached apparent steady states within 15–30 min during the first 30 min of constant exercise (Table 1).

The dynamic characteristics of the SBF and SR in the skin of the inactive upper limb during sinusoidal leg exercise were previously explored to establish the effect of exercise on thermoregulation (Yamazaki et al. 1994; Yamazaki 2002; Yamazaki and Sone 2003). Yamazaki et al. (1994) examined the responses of the body and skin temperatures and forearm SR and showed that the SR phase (~63°) during 4‐min periods of leg cycling always preceded those of the small sinusoidally varying body and skin temperatures. Their subsequent study explored the responses in reflex control between the glabrous and nonglabrous skin of the inactive upper limb (Yamazaki 2002) during leg sinusoidal cycling. The SBF responses in the forearm nonglabrous skin showed a sinusoidal pattern and followed the cyclic changes in WR with a similar phase delay (~70°), whereas the response of palmar glabrous skin was not clearly sinusoidal due to abrupt large fluctuations and sudden irregular reductions. The responses in the forearm SR and SBF in the present study are quite consistent with their results, albeit with some minor differences (i.e., the SR phase delay was greater). These differences may be partly due to differing fitness levels of the subjects, as these responses can be affected by aerobic training status (Yamazaki and Sone 2003). The present results indicated that the dynamics of SBF in the forearm did not explain those of BF–BA. The question remains, however, whether SBF in a glabrous area (i.e., the palm) may play a specific role in the response of BF–BA because the nonglabrous and glabrous SBF responses in the forearm were at least different during exercise (Yamazaki 2002). Furthermore, continuing the constant WR leg exercise, BF–BA [retrograde] returned to baseline, in part, due to cutaneous vasodilation in the forearm and hand (Simmons et al. 2011). Therefore, the relationship between the dynamics of BF–BA (especially the component profiles of antegrade/retrograde BF) and SBF in the glabrous skin during sinusoidal WR leg exercise remains to be fully elucidated.

Another potential candidate is the inactive skeletal musculature in the forearm and hand. It is technically difficult to measure the BF to skeletal musculatures separately from the BF to the skin, except using more invasive methods such as measuring [^125^I] antipyrine clearance. Using this technique, Johnson and Rowell (1975) estimated the relative contributions of skin BF and inactive muscle BF during prolonged constant WR leg cycling and showed that skin and muscle BFs gradually increased and decreased with time, respectively. In addition, indirect estimation by the simultaneous measurement of venous outflows originating mainly from the skin (surface) and musculature (deep) regions indicated that the arterial inflow (i.e., BF–BA) to the upper limb during leg cycling exercise might be diverted to some extent from muscle to the skin (Ooue et al. 2008). Thus, both studies suggest that a larger proportion of the inflow in the conduit artery to the inactive limb is directed to the skin, while this flow is dependent on the thermal environment. It still remain, however, the possibility that the contribution of blood flow to nonexercising muscle beds in forearm is still relatively dominant and induced the antiphasic BF–BA response, because the WR in the present study was relatively low. The future research should therefore examine the relatively higher WR domains.

Recently, Simmons et al. (2011) assessed the BF–BA response during prolonged constant WR exercise and observed a biphasic response in BF–BA during moderate exercise that lasted >30 min. Compared with baseline rest, the BF–BA abruptly decreased within 5 min after the onset of the exercise, but then increased significantly, probably owing at least in part to cutaneous vasodilatation during the prolonged exercise. It was evidenced by the use of an excellent maneuver involving local cooling of the forearm and hand. Their study indicated that the BF patterns assessed during a brief bout of exercise may not always be representative of the responses that may occur during the continuation of exercise, which was then confirmed in other reports (Padilla et al. 2011b; Smith et al. 2014). According to the previously stated result, we hypothesized that phase shift of BF–BA during sinusoidal exercise would be concomitant with that of SBF in the forearm, but could not provide evidence for this. While the reason for this discrepancy has not been precisely determined, it might be due in part to the difference in modes of WR forcing between constant (step) and continuously varied (sine) exercises. Further study is warranted to examine the comparison of the dynamic properties of BF–BA and SBF between step and sinusoidal changes of WR after preceding 30‐min constant exercise to address alternative hypotheses.

In conclusion, the changes in BF into the inactive limb were quite different from the regulation of the circulation and gas exchange (O_2_ transport) to the working muscles during sub‐VT exercise. Although most variables followed the sinusoidal WR fluctuation with similar and predictable delays, BF in the conduit artery to the inactive limb (i.e., BF–BA) demonstrated an approximately antiphasic response, even after a prolonged initial period of constant WR exercise. It appears unlikely that this could be ascribed exclusively to altering the downstream to nonglabrous forearm skin. This finding provides insight into the propagation of endothelial adaptations to the inactive upper limb beyond the active muscles of lower limb exercise.

## Conflict of Interest

No conflicts of interest, financial or otherwise, are declared by the authors.
